# Time-resolving state-specific molecular dissociation with XUV broadband absorption spectroscopy

**DOI:** 10.1126/sciadv.adk1482

**Published:** 2023-11-22

**Authors:** Alexander Magunia, Marc Rebholz, Elisa Appi, Christina C. Papadopoulou, Hannes Lindenblatt, Florian Trost, Severin Meister, Thomas Ding, Michael Straub, Gergana D. Borisova, Junhee Lee, Rui Jin, Alexander von der Dellen, Christian Kaiser, Markus Braune, Stefan Düsterer, Skirmantas Ališauskas, Tino Lang, Christoph Heyl, Bastian Manschwetus, Sören Grunewald, Ulrike Frühling, Ayhan Tajalli, Ammar Bin Wahid, Laura Silletti, Francesca Calegari, Philip Mosel, Uwe Morgner, Milutin Kovacev, Uwe Thumm, Ingmar Hartl, Rolf Treusch, Robert Moshammer, Christian Ott, Thomas Pfeifer

**Affiliations:** ^1^ Max-Planck-Institut für Kernphysik, Saupfercheckweg 1, 69117 Heidelberg, Germany.; ^2^ Ruprecht-Karls-Universität Heidelberg, Grabengasse 1, 69117 Heidelberg, Germany.; ^3^ Leibniz University Hannover, Welfengarten 1, 30167 Hannover, Germany.; ^4^ Deutsches Elektronen-Synchrotron (DESY), Notkestraße 85, 22607 Hamburg, Germany.; ^5^ Helmholtz Institute Jena, Fröbelstieg 3, 07743 Jena, Germany.; ^6^ GSI Helmholtzzentrum für Schwerionenforschung, Planckstraße 1, 64291 Darmstadt, Germany.; ^7^ Center for Free-Electron Laser Science (CFEL), Deutsches Elektronen-Synchrotron (DESY), Notkestr. 85, 22607 Hamburg, Germany.; ^8^ Physics Department, Universität Hamburg, Luruper Chaussee 149, 22761 Hamburg, Germany.; ^9^ J. R. Macdonald Laboratory, Kansas State University, Manhattan, KS 66506,USA.

## Abstract

The electronic and nuclear dynamics inside molecules are essential for chemical reactions, where different pathways typically unfold on ultrafast timescales. Extreme ultraviolet (XUV) light pulses generated by free-electron lasers (FELs) allow atomic-site and electronic-state selectivity, triggering specific molecular dynamics while providing femtosecond resolution. Yet, time-resolved experiments are either blind to neutral fragments or limited by the spectral bandwidth of FEL pulses. Here, we combine a broadband XUV probe pulse from high-order harmonic generation with an FEL pump pulse to observe dissociation pathways leading to fragments in different quantum states. We temporally resolve the dissociation of a specific O_2_^+^ state into two competing channels by measuring the resonances of ionic and neutral fragments. This scheme can be applied to investigate convoluted dynamics in larger molecules relevant to diverse science fields.

## Teaser

The transition from a bound oxygen molecule to state-resolved atoms is followed in time with a combination of XUV-laser sources.

## INTRODUCTION

The measurement of (neutral) fragments and radicals, or chemical shifts within intact molecules, allows us to track molecular dissociation or intramolecular excitation and charge flow (*1*, *2*) on ultrafast timescales (*3*). The understanding of processes in chemically reactive environments, for example, atmospheric or biological processes (*4*–*11*), is facilitated by their sensitivity to electronic structure and atomic sites (*5*–*7*) and the capabilities of time-resolved experiments (*12*). The oxygen molecule is a key component in such environments and its photodissociation is thus an active area of research (*13*–*16*). Tunable extreme ultraviolet (XUV) free-electron lasers (FELs) (*17*) provide a unique tool to trigger electronic or molecular dynamics (*18*) by preparing specific initial states, but mostly lack the spectral bandwidth to detect all relevant resonances and fragments at the same time (*14*, *19*). In contrast, high-order harmonic generation (HHG) pulses (*20*) can be used as XUV broadband probes in transient absorption spectroscopy (*21*) to simultaneously detect several neutral and ionic fragments and chemical shifts within a molecule, but up to now have only been used in combination with pump pulses at lower (optical) frequencies (*22*), and thereby lose the benefit of a state- and/or site-specific excitation.

Here, we combine the benefits of the FEL pump and HHG probe pulses in all-XUV transient absorption spectroscopy to specifically address and clock the fragmentation of the $O_{2}^{+}(c^{4}{\text{Σ}}_{u}^{-}\;v=0)$ state by fragment-tunneling through a nuclear potential energy well and predissociation. Theoretical estimates for the timescale of this process are unsettled (*23*–*27*) because of the high sensitivity of the underlying tunneling process on the energy barrier, with no direct time-resolved measurement so far. We thus perform this experiment also as a benchmark to illustrate how our experimental approach can be used to track tunneling and predissociation and, in general, dissociation dynamics of ionic and neutral fragments, including their final quantum state, on ultrafast timescales, which can be straightforwardly applied to more complex molecules (*28*). Understanding tunneling processes of charge carriers—on its own or in competition with other charge flow dynamics—can be relevant in many research areas and applications, from the production of attosecond pulses via HHG (*29*–*31*), to properties of transistors (*32*), semiconductors (*33*) or two-dimensional materials (*34*), as well as in chemical reactions (*4*, *5*, *7*, *35*) and charge transfer in proteins.

## RESULTS

**Fig. 1.**
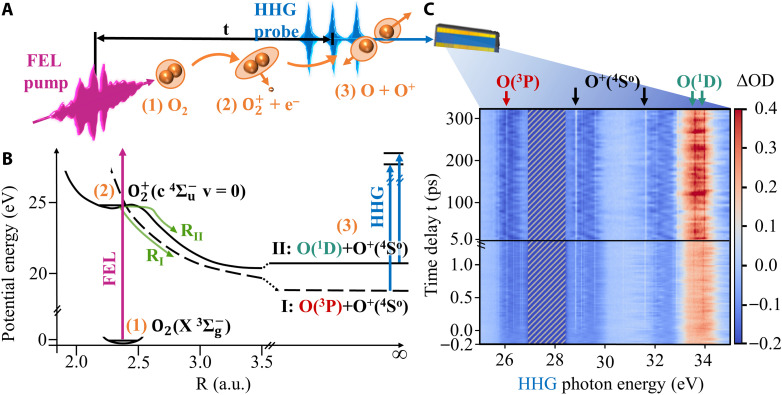
Overview of the measurement concept and absorption data. (**A**) Experimental scheme. The FEL pulse excites the oxygen molecule from its ground (1) to the excited state in the molecular ion (2), from where it dissociates. After a delay time *t*, the HHG pulse (train) probes the resulting fragments (3), allowing to identify fragments resulting from the targeted c ${}^{4}{\text{Σ}}_{u}^{-}$ state. (**B**) Scheme of relevant potential-energy curves of oxygen based on (*36*). The excited state can couple to another state (dashed line) and thereby predissociate into the (I) dissociation channel (lower green arrow). In addition, being confined by a potential-energy barrier, tunneling through that barrier into the (II) dissociation channel (upper green arrow) is possible. (**C**) Time-resolved differential absorbance Δ*OD*(*t, E*_HHG–ph._) for 27.7 eV FEL photon energy. For positive time delays, the FEL pulse arrives first. The data are compiled from a femtosecond and a picosecond scale measurement, the change of the time-delay axis is indicated with the black horizontal line. The sharp absorption features, arising on a timescale of femtoseconds (lower half) and changing their intensity over hundreds of picoseconds (upper half), are identified as different resonant electronic transitions in various fragments. The sharp resonances relevant to the dissociation process are marked in red for O(^3^P), black for O^+^(^4^S^o^), and cyan arrows for O(^1^D) fragments. Broader features are due to the residual HHG spectral structure (cf. Fig. 3). The area around 27.7 eV is overlaid with gray stripes because the residual FEL stray light is more intense than the harmonics and thus precludes a meaningful measurement of HHG spectra in this spectral region. a.u., atomic units.

In the experiment (illustrated in Fig. 1A), we use an FEL pulse at 27.7-eV central photon energy and ~0.3-eV bandwidth, which is well above the resonance energy of 24.6 eV of the excited molecular $O_{2}^{+}(c^{4}{\text{Σ}}_{u}^{-}\;v=0)$ state relative to the ground state of the neutral O_2_ molecule (for simplicity, we will refer to this specific state of $O_{2}^{+}$ as “excited molecular state” below). This ensures that the whole FEL spectrum contributes to the molecular excitation process, where the residual energy is taken away by the emitted electron. The excited molecular state can predissociate via nonadiabatic couplings to other states into the first dissociation channel of oxygen: O(^3^P) + O^+^(^4^S^o^) (*36*), or dissociate via tunneling into the second dissociation channel: O(^1^D) + O^+^(^4^S^o^) (*36*) (Fig. 1B). The broadband (Δω ~ 20 eV) HHG probe pulse—after transmission through the sample—allows to detect the difference in spectral absorption Δ*OD* caused by emerging fragments in different states (more details can be found in Materials and Methods). By scanning the time delay t between the HHG and FEL pulses, first with 20-fs steps for delays <1.5 ps and afterward with coarser 5-ps steps, we stepwise record a time-dependent differential absorbance Δ*OD*(*t, E*_HHG–ph._) (see Fig. 1C). The spectrally sharp absorption features originate from resonant transitions within the neutral and ionic fragments produced in various excited atomic states after the dissociation of the excited molecular state. We are able to identify resonances that are attributed to the first dissociation channel with O(^3^P) at 26.1 eV (*37*) (red labeled) as well as to the second dissociation channel with O(^1^D) at 32.9 and 33.9 eV (*38*) (cyan), whereas O^+^(^4^S^o^) at 28.8 and 31.6 eV (*39*, *40*) (black) appears in both channels. The rise in amplitude of the resonances along the time-delay axis (Fig. 1C) is a measure of the time-resolved abundance of the corresponding fragment.

In the following, we extract the dissociation times of the two channels I and II by using a rate-equation model: The population in the excited molecular state *N*_0_(*t*) dissociates into the first dissociation channel with population *N*_I_(*t*) and rate *R*_I_ or, alternatively, into the second channel with population *N*_II_(*t*) and rate *R*_II_. Solving the differential equations (see Materials and Methods) leads to the following exponential solutions of the population dynamics of the fragments

$$N_{O+(4S)}(t)=N_{I}(t)+N_{II}(t)=1-\exp[-(R_{I}+R_{II})t]$$ (1.1)

$$N_{O(1D)}(t)=N_{II}(t)=R_{II}/(R_{I}+R_{II})\;\{1-\exp[-(R_{I}+R_{II})t]\}$$ (1.2)

$$N_{O(3P)}(t)=N_{I}(t)=R_{I}/(R_{I}+R_{II})\;\{1-\exp[-(R_{I}+R_{II})t]\}$$ (1.3)

As a result, all three fragments appear with the same exponential time constant

$$\tau_{d}=1/(R_{I}+R_{II})$$ (2)

through dissociation of the excited molecular state, which we thereby define as the dissociation time. In contrast, the amplitudes and hence probabilities of the three fragments in Eqs. 1.1 to 1.3 are different. To verify whether this is the process that leads to the observed experimental features, we fit lineouts of the measured resonance lines *r* for a given fragment *f* at a specific HHG photon energy interval centered at *E_r_* with exponential functions *A_f_*(*E_r_*)(1 − exp[−*t*/τ*_f_*)] (see Materials and Methods).

**Fig. 2.**
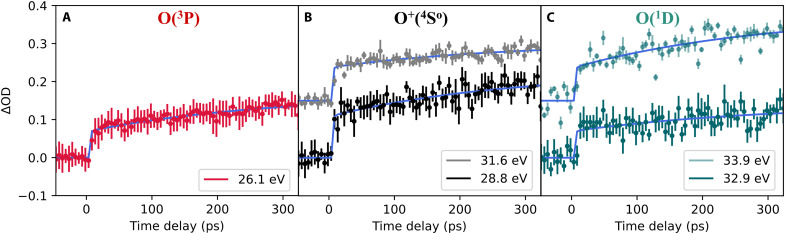
Time-resolved, background-corrected resonant Δ*OD*(*t*) lineouts (dots with error bars) and corresponding fits (blue lines). Lineouts and fits are shown for (**A**) O(^3^P), (**B**) O^+^(^4^S^o^) and (**C**) O(^1^D) fragments. In (B) and (C), two resonances at different HHG photon energies (see legends) are presented with different colors. These are fitted with a global fit to extract a single dissociation time constant per fragment, but with different amplitudes, due to different cross sections. The resonances shown in fainter colors are shifted upward by 0.15 along the Δ*OD* axis for better. visibility. Error bars represent the SD of data points after averaging over 400 individually calculated Δ*OD*s for every time step and resonance lineout.

Figure 2 shows the lineouts of the Δ*OD*(*t*) for these resonances along the time-delay axis, together with corresponding exponential fits. Off-resonant backgrounds have been subtracted for each lineout as discussed in Materials and Methods. The sharp rises in Δ*OD* for all resonance lineouts around time zero is a combined effect of all faster (femtosecond to single picosecond) dissociation processes, which cannot be resolved on the coarser 5-ps delay step scale in this measurement (cf. Materials and Methods). Notably, the $O_{2}^{+}(c^{4}{\text{Σ}}_{u}^{-})$ state supports a second, higher-lying vibrational level, *v* = 1, which dissociates via tunneling much faster than the *v* = 0 level, since it faces a lower potential-energy barrier. The *v* = 1 tunneling dissociation is expected to take place on a femtosecond timescale (*24*, *25*), which is not resolved in the present experiment. In addition, these processes can be attributed to $O_{2}^{+}(B^{2}{\text{Σ}}_{g}^{-})$ dissociation into the first dissociation channel (*41*), and two pump-photon processes and respective O^+^+O^+^ dissociation (*14*), which are all known to dissociate faster than our 5-ps resolution. For all resonance lines, we find this fitted timescale to be τ_fast_ ≲ 1 ps. In view of the orders-of-magnitude slower dissociation process of the here-targeted $O_{2}^{+}(c^{4}{\text{Σ}}_{u}^{-}\;v=0)$ excited molecular state, the subpicosecond resolution is not required for its scrutiny. The resulting fit parameters, dissociation times τ_*f*,slow_ and resonance amplitudes *A_r_*, are shown in Table 1.

**Table 1.** Fit parameters quantifying the tunneling and predissociation dynamics of the O^+^_2_ (c^4^Σ^-^_u_ *v* = 0) state. *A_r_* is the amplitude of the relative optical density Δ*OD* of resonance *r* of a given fragment *f* at large time delays. τ_*f*,slow_ designates the exponential rise time at which fragment *f* appears via tunneling [O(^1^D) and O^+^(^4^S^o^)] and predissociation [O(^3^P) and O^+^(^4^S^o^)]. arb.u., arbitrary units.

Fragment *f*	O(^3^P)	O^+^(^4^S^o^)	O(^1^D)
**τ_*f*,slow_** **(ps)**	280 ± 120	290 ± 170	280 ± 200
***A**_**r**_* **(arb.u.)**	*A*_26.1eV_ = 0.097 ± 0.022	*A*_28.8eV_ = 0.119 ± 0.037 *A*_31.6eV_ = 0.067 ± 0.022	*A*_32.9eV_ = 0.067 ± 0.028 *A*_33.9eV_ = 0.138 ± 0.052

The three independently fitted values of τ_*f*,slow_ agree well within their error bars and support the model of the dissociation process from a single reservoir, the excited molecular state, into two channels, the two dissociation limits, with different probabilities but the same time constant, as described with the rate-equation model above. Averaging over the three individual τ_*f*,slow_ from Table 1, we extract the tunneling and predissociation time of the excited molecular state as $\bar{\tau }=280\pm 160\;\mathrm{ps}$. This value lies within the range of theoretical estimations, which span from a few picoseconds to 10 ns (*23*–*27*). Furthermore, measurements of the spectral linewidth of the $O_{2}^{+}(c^{4}{\text{Σ}}_{u}^{-}\;v=0)$ state (*27*, *42*) have estimated a lower limit for the dissociation time of a few picoseconds, but their spectral resolution would not have allowed to infer a lifetime of 280 ps, especially because potential rotational broadening was not resolved. Thus, our time domain–based result is compared with their spectral domain–based findings a more direct assignment of the dissociation time. The ratio of the two rates *R*_II_/*R*_I_ equals the ratio of the probabilities *P*_II_/*P*_I_ of the excited molecular state for dissociation into the first or second dissociation channel, which is known from previous studies (*36*) to be *P*_II_/*P*_I_ = 1.5. With the help of this ratio and the dissociation time $\bar{\tau }$, we determine the individual rates of dissociation into the two channels I and II: *R*_I_ = 1.4 ± 0.8 ns^−1^, *R*_II_ = 2.1 ± 1.2 ns^−1^. More details are provided in Materials and Methods. To the best of our knowledge, this provides the first direct time-resolved measurement of the dissociation time and the individual rates. Comparable experiments (*16*, *43*) are complementary to our results, studying the neutral Rydberg series converging to the $O_{2}^{+}(c^{4}{\text{Σ}}_{u}^{-})$ ionic state, which in parallel to predissociation also autoionizes on a faster timescale, but not the $O_{2}^{+}(c^{4}{\text{Σ}}_{u}^{-})$ state itself.

## DISCUSSION

These results illustrate how state-specific ultrafast molecular dynamics can be extracted with spectrally and temporally resolved FEL pump–HHG probe transient absorption spectroscopy. In particular, the use of HHG probe pulses at an FEL facility with a spectral bandwidth much (>10 times) broader than the average FEL pulse bandwidths is essential to detect both neutral and ionized fragments. Thereby, we gain insight into the state-specific molecular breakup including experimentally distinguishing both competing dissociation channels and determine its dissociation time, which is strongly influenced by the interplay of the parallel tunneling and predissociation channel. In the future, this scheme can be applied to molecular systems, allowing both precision tests of state-of-the-art quantum dynamics theory in small molecules (*44*–*46*) as well as time-resolving state-specific molecular dynamics in more complex systems with a broad dynamic range from nanoseconds to femtoseconds. It will be possible to experimentally address questions about intermediate states or electronic changes faster than or in interplay with structural dynamics (*47*). Furthermore, electronic charge transfer within intact neutral molecules can be investigated, extending previous studies (*2*, *48*, *49*) to a higher (XUV) photon energy or to neutral and more complex systems covering several atomic sites, respectively. Our approach provides a complementary method to charged-particle–based detection schemes, which, in addition, can be operated in parallel in the same experimental setup using a reaction microscope (REMI) (*50*). It suggests further avenues for unraveling and manipulating element-selective ultrafast molecular dynamics and the coherent control of atoms and molecules (*51*) with XUV/x-ray multipulse sequences, involving the unique combination of tunable and intense (*52*, *53*) FEL pump pulses and spectrally broad HHG-based probe pulses. Extensions of the here-demonstrated technique hold promise to promote diverse fields of science. For example, in radiation chemistry, where chemically active fragments and radicals interact with the environment, precise knowledge of the involved electronic configurations of all product states is relevant for understanding complex interactions and chemical cycles. Similarly, biochemical reactions with charged and neutral fragments are research areas that may benefit from the transient-absorption technique that we examined and benchmarked here for a prototypical small molecule.

## MATERIALS AND METHODS

### Experimental setup

This experiment was performed at the FL26 beamline of the free-electron laser FLASH, DESY, Hamburg. The REMI permanent end station, described in more detail in (*50*, *54*, *55*), has recently been upgraded by adding an absorption setup with an XUV spectrometer, which allows us to detect XUV spectra with a resolution of ~30 meV (*56*). In addition, a beamline for HHG driven by the output of the FLASH2 pump-probe OPCPA (optical parametric chirped-pulse amplification) laser system has been integrated into the FL26 beamline (*57*–*59*). By using the master timing system of the FLASH facility, the OPCPA system is synchronized with the FEL and produces the same pulse pattern. The optical delay stage of the driving laser system can introduce up to 4 ns of time delay between the HHG and FEL pulses with femtosecond precision, allowing to study the dynamics of processes over a wide time range. After suppression of the remaining driving radiation by a 100-nm-thick Al filter, the HHG pulses are coupled into the FLASH2 beamline by means of a motorized hyperboloidal mirror, described in more detail in (*57*, *58*). From this point, the HHG beam propagates parallel to the FEL beam, shifted upward by around 7.5 mm. We focus the FEL and HHG beams with an ellipsoidal mirror into the REMI (*50*) and further downstream we refocus both beams with a toroidal mirror into the interaction region. There, we spatially overlap them by adjusting the HHG in-coupling mirror with the help of a phosphorus screen imaged by a charge-coupled device (CCD) camera.

**Fig. 3.**
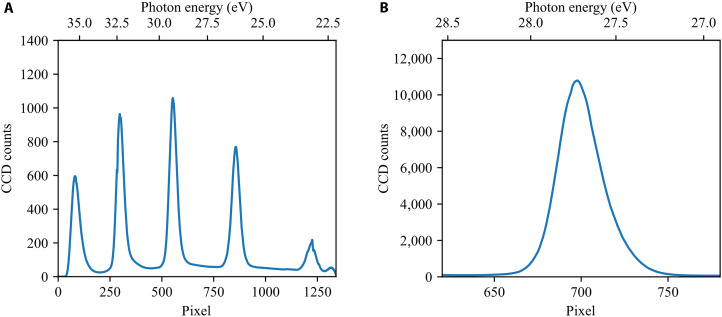
HHG and FEL reference spectra. (**A**) Average of all HHG reference spectra of static molecular oxygen absorption recorded during the time-delay scan without the FEL pump pulse. (**B**) Average of all FEL reference spectra recorded during the time-delay scan as stray light. The static molecular oxygen absorption is nonresonant and flat in this confined spectral regime, thus mainly decreasing the overall FEL intensity without major spectral changes.

For the here presented experiment, the FEL pulses are spectrally centered at 27.7 eV and have a time duration of 100 fs (full width at half maximum, FWHM), as estimated by an electron-bunch duration measurement before the beamtime. The pulse energy of the FEL pulses was measured to be 37.3 ± 4.1 μJ before the FL26 beamline. Taking the beamline transmission of 29% into account, we estimate an FEL pulse energy of 10.7 ± 1.2 μJ at the oxygen target. The HHG pulses are generated by focusing the OPCPA driving pulses with ~15-fs duration (FWHM) and 780-nm central wavelength into a gas cell filled with 100 mbar of Kr. The pulse duration of the HHG pulses was not measured directly but is expected to be shorter than the driving pulse duration due to the nonlinear nature of HHG production. The HHG pulse energy was estimated at ~100 pJ at the generation point. Taking into account the combined transmission of a 100-nm-thick aluminum filter and the incoupling mirror, as well as the residual beamline transmission, we estimate 18 pJ at the oxygen target. Figure 3 shows averaged reference spectra of the HHG pulses and FEL pulses (as stray light) recorded during the time-delay scan shown in Results. In the interaction region of the transient-absorption setup, a cell of 3-mm length with 200-μm holes on both sides along the beam axis is filled with oxygen at 8 mbar backing pressure and placed in the overlapping focus of both beams. The focal diameter of the FEL beam at the target interaction was not directly measured in this experiment, but previous measurements at this beamline resulted in a focal diameter of 5 to 10 μm (*56*). Behind the interaction region, the FEL beam is spatially separated from the HHG beam allowing it to block the FEL beam with an aluminum plate. We minimize the remaining FEL stray light with an additional 100-nm-thick aluminum filter to avoid saturation of the CCD detector, simultaneously attenuating the measured HHG beam only by ~80%. However, this still leaves us with a spectral region around the central FEL photon energy of 27.7 eV, where we cannot record HHG spectra (see Fig. 1C). We disperse the HHG pulses with a Hitachi variable line-space grating and record the resulting XUV spectra with a PIXIS XUV-sensitive CCD camera. Both HHG and FEL are produced in the FLASH pulse train mode consisting of pulse trains with a 10-Hz repetition rate, which contain 38 pulses with a spacing of 10 μs. We find the temporal overlap between FEL and HHG pulses by measuring the transient absorption spectra of argon, where the ionization and thereby creation of ionic resonances in the HHG spectra takes place orders of magnitude faster (in subfemtoseconds) than the experimental temporal jitter (tens of femtoseconds). We record the HHG spectra with 10-Hz and 10-ms exposure time, thereby integrating over the pulse train of 38 HHG spectra.

### Recording of Δ*OD* and extraction of resonance lineouts

Using the FL26 beamline fast shutter, we can block every second FEL pulse train, thereby alternating between measuring absorption spectra *I*(*E*_HHG–ph._) and static (unpumped) molecular oxygen reference spectra *I*_0_(*E*_HHG–ph._) at 5 Hz. We average over all *I*(*E*_HHG–ph._) and *I*_0_(*E*_HHG–ph._) for a given time delay, respectively, and calculate the Δ*OD*(*E*_HHG−ph._) via

$$\text{Δ}OD(E_{\mathrm{HHG}-\mathrm{ph}.})=-\log_{10}\left[\frac{\bar{I}(E_{\mathrm{HHG}-\mathrm{ph}.})}{{\bar{I}}_{0}(E_{\mathrm{HHG}-\mathrm{ph}.})}\right]$$ (3)

By using the delay stage for the HHG-driving IR pulse, we scan the time delay *t* between the HHG and FEL pulses from −200 to 1400 fs in steps of 20 fs and additionally from −46.5 to 323.5 ps in steps of 5 ps. In this way, we record the time-dependent Δ*OD*(*t, E*_HHG-ph._) with the two time-delay scales in Fig. 1C). We record 1000 frames at a given time-delay position for the scan with femtosecond resolution and with 400 frames for the picosecond scan. By comparing Δ*OD* spectra of late time delays with early delays, we identify spectral regions of resonant transitions in the fragments and define all other spectral regions as off-resonant backgrounds. For every resonance, we average over 1 to 4 pixels, both for the resonance itself as well as for a nearby off-resonant spectral region, spanning ~10 to 40 meV. By subtracting the nearby off-resonant lineout from the resonant one, we ensure to account for the resonant effects of the fragments without off-resonant residual absorption changes. These lineouts along the time-delay axis (of the picosecond-resolution scan) are shown in Fig. 2. Fitting two off-resonant lineouts with a complementary error function, erfc[(*t*-*t*_0_)/σ], we find the temporal resolution to be around 300 fs [cf. the “Fitting procedure of resonant lineouts Δ*OD*(*t, E_r_*)” section], which gives an estimate of the combined effects of (i) both individual FEL and HHG pulse durations and (ii) the temporal jitter and drifts between the pulses. Potential electronic configuration changes during fragmentation, which would lead to shifts in the measured resonance positions, are expected to be on the same order of magnitude or faster (*14*) and thus cannot be resolved in this experiment. In addition, the position *t*_0_ of the complementary error function allows for an in situ determination of the temporal overlap of FEL and HHG pulses.

### Differential rate–equation model

The dissociation process of the excited molecular state given in Results is governed by the following set of differential rate equations

$$\begin{array}{rl} & \frac{{dN}_{0}(t)}{dt}=-R_{I}N_{0}(t)-R_{II}N_{0}(t);\frac{{dN}_{I}(t)}{dt}=R_{1}N_{0}(t);\\ & \frac{{dN}_{II}(t)}{dt}=R_{II}N_{0}(t);N_{0}(t=0)=1 \end{array}$$ (4)

Their exponential solutions and the resulting exponential dynamics of the individual fragments are given in Results.

### Fitting procedure of resonant lineouts Δ*OD*(*t*, *E_r_*)

For all three fragments *f*, all resonances *r* are fitted simultaneously with the following equation

$$\begin{array}{rl} \text{Δ}OD(t,E_{r}) & =\exp\left[-\frac{{\left(t-t_{0}\right)}^{2}}{2\sigma^{2}}\right]*[\theta (t,t_{0})(A_{f,\mathrm{slow}}(E_{r})\{\\ & \;1-\exp\left[-\frac{\left(t-t_{0}\right)}{\tau_{f,\mathrm{slow}}}\right]\}+A_{f,\mathrm{fast}}(E_{r})\{\\ & \;1-\exp\left[-\frac{\left(t-t_{0}\right)}{\tau_{f,\mathrm{fast}}}\right]\})]+b(E_{r}) \end{array}$$ (5)

where the two amplitudes *A_f,d_* (*d* = slow, fast) are energy-dependent contributions of the different dynamics *d* leading to the same fragment and the two τ*_f,d_* are the time constants of the corresponding dynamics; *b*(*E_r_*) is a time-independent offset; *t*_0_ is the temporal overlap position; θ(*t*, *t*_0_) is a Heaviside function, and $\exp\left[-\frac{{\left(t-t_{0}\right)}^{2}}{2\sigma^{2}}\right]*(\ldots )$ represents the convolution of the molecular dynamics with the temporal instrument response function, here chosen to be a Gaussian function with SD σ. The *t*_0_ and σ parameters are determined by independent fits to off-resonant regions of the Δ*OD*, as described in the “Recording of Δ*OD* and extraction of resonance lineouts” section. The second exponential function indexed with “fast” is necessary to describe the sharp rises in Δ*OD* for all resonance lineouts around time zero as described in Results. For all resonance lines, we find this timescale to be τ_fast_ ≲ 1 ps, which can be extracted by a similar fitting procedure from the femtosecond-scale measurement more precisely as τ_fast_ ≲ 300 ± 100 fs. It is negligible for the much slower dissociation of the $O_{2}^{+}(c^{4}{\text{Σ}}_{u}^{-}\;v=0)$ state.

### Extracting the dissociation rates *R*_I_ and *R*_II_

With the help of the dissociation time $\bar{\tau }$ of the $O_{2}^{+}(c^{4}{\text{Σ}}_{u}^{-}\;v=0)$ state, the ratio of the rates (*36*) *R*_II_/*R*_I_ = 1.5, and Eq. 2, the individual rates *R*_I_ and *R*_II_ can be estimated as follows

$$R_{I}=1/\bar{\tau }-R_{II}=1.4\pm 0.8\;{\mathrm{ns}}^{-1}$$ (6)

$$R_{II}=1/\bar{\tau }-R_{I}=2.1\pm 1.2\;{\mathrm{ns}}^{-1}$$ (7)
